# Everolimus in Invasive Malignant Renal Epithelioid Angiomyolipoma

**DOI:** 10.3389/fonc.2020.610858

**Published:** 2021-01-26

**Authors:** Gang Guo, Liangyou Gu, Xu Zhang

**Affiliations:** Department of Urology, The Third Medical Centre, Chinese PLA General Hospital, Beijing, China

**Keywords:** renal epithelioid angiomyolipoma, everolimus, tuberous sclerosis complex, mutation, next-generation sequencing

## Abstract

**Background:**

To evaluate the efficacy and safety of everolimus, a mTOR inhibitor, on invasive malignant renal epithelioid angiomyolipoma (EAML).

**Materials and Methods:**

From Oct 2014 to May 2019, we collected data from seven patients with a definite (clinical and pathological) diagnosis of EAML received everolimus in our hospital. Targeted sequence capture array technique with next-generation of high throughput sequencing (NGS) were performed to detect mutations of TSC1/2 genes. All patients had received surgery and everolimus. The clinical efficacy and safety of the therapy were evaluated.

**Results:**

Mutations of TSC1 and TSC2 were detected in two and three patients though targeted sequence capture array technique with NGS, respectively. Among seven patients, three had missense mutations, one had nonsense mutation, and one had the large fragment deletion mutation. Five patients accompanied with tuberous sclerosis complex (TSC) were identified. All patients were administered 10mg everolimus once daily, the treatment duration lasted for 3 to 28 months. The objective response was assessed 3 months later, five partial response, two stable disease (SD), the mean greatest tumor diameter of all patients decreased from 9.6 to 5.2cm. Six patients stayed SD and one patient died during follow up. Patients accompanying with TSC had better responses to everolimus compared with non-TSC.

**Conclusion:**

The mTOR inhibitor can be an effective treatment for patients with invasive malignant renal EAML. Patients with TSC may benefit more from the therapy.

## Introduction

Angiomyolipoma (AML) is one of the most common benign solid tumors in the kidney (1). Histologically, it is composed of different proportions of fat, blood vessels, and smooth muscle (2). However, epithelioid angiomyolipoma (EAML), is an uncommon subtype of renal AML, has been defined as a mesenchymal neoplasm derived from perivascular epithelioid cells (3, 4). Unlike typical AMLs, these lesions are usually predominantly epithelioid and demonstrate little or no macroscopic fat (5). It was reported that patients with EAML were significantly younger compared to AML (4). The reported malignant potential and high-aggressive biologic behaviors of renal EAML, as local recurrence, distant metastasis or death, were primarily based on case reports (6).

The standard treatment for EAML is still unknown at present, surgical resection is effective, and complete tumor resection can improve the outcome. Moreover, Kenerson et al. (7) reported abnormal activation of mTOR pathway may contribute to renal EAML growth and progression. In some patients, mTOR inhibitors such as rapamycin and everolimus reduced the size of the tumors, suggested that mTOR inhibitors may be therapeutic option for EAML. In this study, we described our experience of the comprehensive therapy including surgery and everolimus in seven patients with invasive malignant renal EAML. This comprehensive therapy strategy showed great clinical efficacy, which reflected the potential benefit for invasive malignant renal EAML.

## Materials and Methods

The study was approved by Medical Ethics Committee of our hospital, and informed consent was acquired from each patient. The protocol for this study was registered on Chinese Clinical Trial Registry (ChiCTR-OPN-16008236).

### Patients

From October 2014 to May 2019, seven patients with invasive malignant renal EAML were treated in our center, the related data were extracted from a prospectively maintained database. Invasive malignant renal EAML was defined as patients with renal EAML experienced recurrent or metastatic disease. Clinical presentations, tumor size, pathological and radiological features, surgical methods, and follow-up information were recorded.

### TSC1/2 Next-Generation Sequencing

Five milliliters of venous blood was drawn from subjects and controls, and genomic DNA was extracted by standard procedure (QIAamp DNA Blood Midi Kit, Qiagen, Hilden, Germany). Then the purified DNA fragments were subjected to an end repair, an addition of “A” and adapter reactions. Finally, the DNA database of individuals was established.

After obtaining raw reads from the Illumina Pipeline software (version 1.3.4), we started to analyze those. Firstly, evaluating the quality of sequencing, and remove low quality reads and reads contaminated by adapters. Then, performing sequence alignment by the BWA software (Burrows Wheeler Aligner) and HG19. In the meanwhile, evaluating the efficacy of sequence capture, and inquiring SNV (single nucleotide variant) and Indel (insertion and deletion) by SOAPsnp and SAMtools. Finally, generating target region base polymorphism results, comparing databases (NCBI dbSNP, HapMap, 1000 human genome dataset, and database of 100 Chinese healthy adults), and annotating and screening the suspicious mutations founded.

### Everolimus Treatment and Evaluation

After the first operation, these seven patients received everolimus when disease recurrence or progression. All patients were treated with everolimus (Novartis Pharmaceuticals, Switzerland) at an initial dosage of 10 mg/day QD orally until disease progression or grade III/IV drug-related adverse events which requiring reduction or discontinuation. The clinical response, plasma drug concentration, and adverse events were collected regularly.

All patients performed spiral CT or MRI for baseline tumor lesion before receiving everolimus. The largest tumor was recognized as the target lesion. The response is evaluated by solid tumor’s effect evaluation criterion (RECIST version 1.1) (8) at 1, 3, 6, 12 months, and then every 6 months. Adverse events were evaluated every cycle and graded according to the National Cancer Institute Common Terminology Criteria for Adverse Events (NCI CTCAE 4.0, May 2009) (9). The plasma concentration of everolimus was determined by measuring the serum concentration of rapamycin and converted with the reported formula (10).

## Results

### Clinical and Pathological Data

For all patients, three were male and four were female, and the median age was 35 (17–60) years. Five patients firstly presented with symptom of low back pain and other two with renal tumor identified by ultrasound during physical examination, and one of those patients accompanied with hematuria. The invasive renal EAML in four patients were unilateral on left side, other three were bilateral, two patients diagnosed as tuberous sclerosis by clinical while three patients diagnosed as tuberous sclerosis by gene test. Five patients underwent radical nephrectomy, and two underwent partial nephrectomy.

All of the seven patients had disease progressed after the first operation (7–100 months). The median time to disease progression was 25.8 months. Three had local recurrence and four had multiple metastases; two relapsed patients underwent a secondary surgical resection, and one patient with distant metastases underwent a resection of the right scapula metastases.

**Figure 1:**
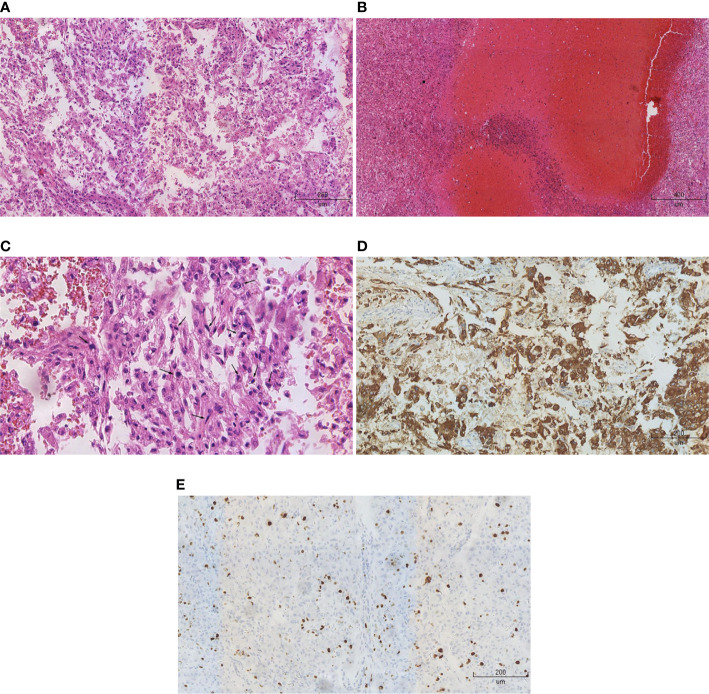
Pathological and immunohistochemical analysis. **(A)** HE staining (x10) showed that epithelioid cells were predominant in the tumors, but adipose tissue was insufficient. **(B)** HE staining (x5) showed a large number of necrotic lesions in the tumor tissues. **(C)** HE staining (x20) showed polymorphonuclear and heterokaryon tumor cells. **(D)** Immunohistochemistry (x10) showed that HMB45 was positive. **(E)** Immunohistochemistry (x10) showed that the rate of positive-Ki-67 was 20%.

All patients were pathologically diagnosed as malignant epithelioid renal angiomyolipoma and positive HMB45 staining. The positive rate of Ki-67 was more than 10% and mitotic figure was more than 2/10 HPF in five patients (**Figure 1**). For the other two patients, the positive rate of Ki-67 was 3% and the mitotic figure was 1–2/10 HPF (**Table 1**).

**Table 1:** Clinical and pathological data of included patients.

	Case 1	Case 2	Case 3	Case 4	Case 5	Case 6	Case 7
Age/gender	34/male	35/female	50/male	60/female	27/female	35/male	17/female
Initial site	Left	Bilateral	Left	Bilateral	Bilateral	Left	Left
Symptoms	Hematuria	Backache	Backache	Backache	Abdominal distension	Abdominal distension and pain	Backache
Maximum tumor diameter (cm)	15	9	10	12	14	9	9
T stage	T3a	T2b	T2b	T2a	T3a	T3a	T2b
Type of surgery	Radical	Partial	Radical	Radical	Partial	Radical	Radical
Progression after surgery (month)	7	10	100	12	6	20	6
Site of recurrence or metastasis	Local recurrence, liver, and lung	Right kidney	Scapula, lung	Local recurrence	Local recurrence, abdominal cavity	Left kidney area, liver	Bone, spleen
Re-operation	Recurrent lesion resection	No	Metastatic lesion resection	Partial right nephrectomy	No	No	No
Maximum diameter of recurrence or metastasis lesions after everolimus (cm)							
Baseline	10	5	4	10	8	15	4
1 month	7	3	2	8.5	4	6	4
3 months	7	1.5	0.7	8	3	5	4.5
6 months	6	1	0.5	7.5	3	6	–
12 months	9	1	0.5	7.5	3	–	–
Follow-up (month)	28	18	18	12	12	6	3
Progression-free survival (month)	16	18	18	12	12	6	3
Survival or not	No	Yes	Yes	Yes	Yes	Yes	Yes
Mitotic figure/HPF	2–3/10	1–2/10	3–4/10	1–2/10	3–4/10	2–3/10	3–4/10
HMB45 staining	Positive	Positive	Positive	Positive	Positive	Positive	Positive
Ki-67	20% positive	3% positive	30% positive	3% positive	20% positive	10% positive	20% positive

### TSC1/2 Next-Generation Sequencing

Sequence capture by the BWA software shows that the average base coverage of all NGS samples was 99.88%, and the minimum sample coverage was 99.44%. The average proportion of targeted area with average sequencing depth > 30X loci was 98.92%, the minimum value was 97.35%. The average sequencing depth of all samples was 280.4X, and the minimum sequencing depth was 168.4X. Moreover, the average and the median sequencing depth of each exon of TSC1 and TSC2 gene were similar, which indicated that the target sequence capturing next-generation sequencing had great randomness.

A total of two clinically meaningful TSC2 mutations and 2 TSC1 mutation were detected, including three missense mutations, one large fragment deletion mutation; one had point mutations without clinical meaning; two did not have mutations (**Table 2**). According to the previous literatures and the LOVD database MJ (11), c.1700C > T missense mutation, a mutation unconfirmed clinical meaning, in exon 15 of TSC1 was detected in case 1. The routine Sanger sequencing was used to verify the five NGS detected mutation sites in seven cases. The results were identical with those of target sequence capture sequencing, and the coincidence rate was 100%.

**Table 2:** next-generation sequencing results of TSC1/2 in patients.

	Case 1	Case 2	Case 3	Case 4	Case 5	Case 6	Case 7
Gene	TSC1	TSC2	TSC2	TSC1	–	TSC2	–
Subregion	EX15/CDS13	EX27 EX41	EX17/CDS16	UTR3	–	EX2-3	–
Chromosomal location	chr9:135781265	chr16:2107157	chr16:2120572	chr9:35767943	–	chr16:2038155-2078531	–
Protein change	p.Ala567Val	p.Gly1001Glu	p.Arg611Gln	–	–	–	–
Nucleotide change	c.1700C>T	c.3002G>A	c.1832G>A	c.3679G>A	–	–	–
Mutation type	Missense	Missense	Nonsense	Missense	–	Large fragment deletion	–
Novel	reported	reported	reported	reported	–	reported	–
ACMG	VUS	like pathologic	like pathologic	VUS	–	like pathologic	–

VUS, variants of uncertain significance.

### The Efficacy and Safety of Everolimus

**Figure 2:**
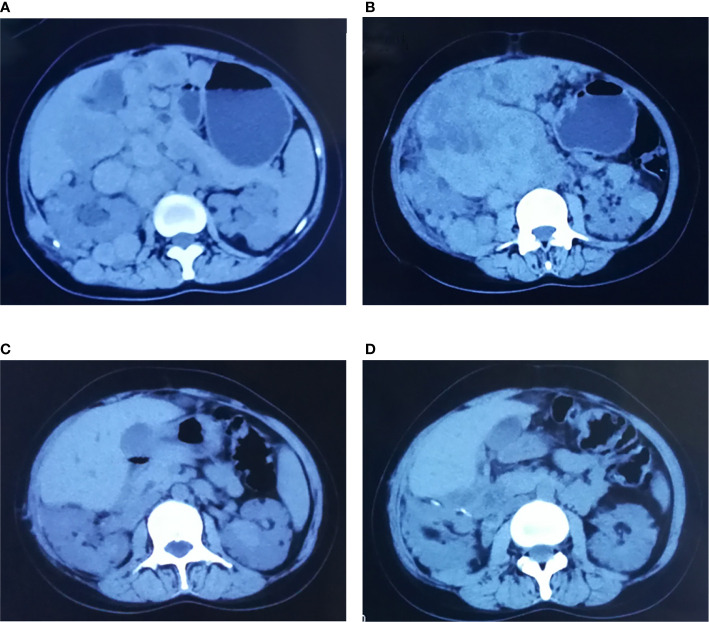
Comparisons of CT before and after treatment with everolimus (case 5). **(A, B)** Before the treatment, CT scan: right retroperitoneal and abdominal cavity multiple space occupied lesion. **(C, D)** After 3-month treatment of everolimus, CT scan: space-occupying lesions in retroperitoneal and abdominal cavity mostly disappeared, the lesions shrank significantly.

**Figure 3:**
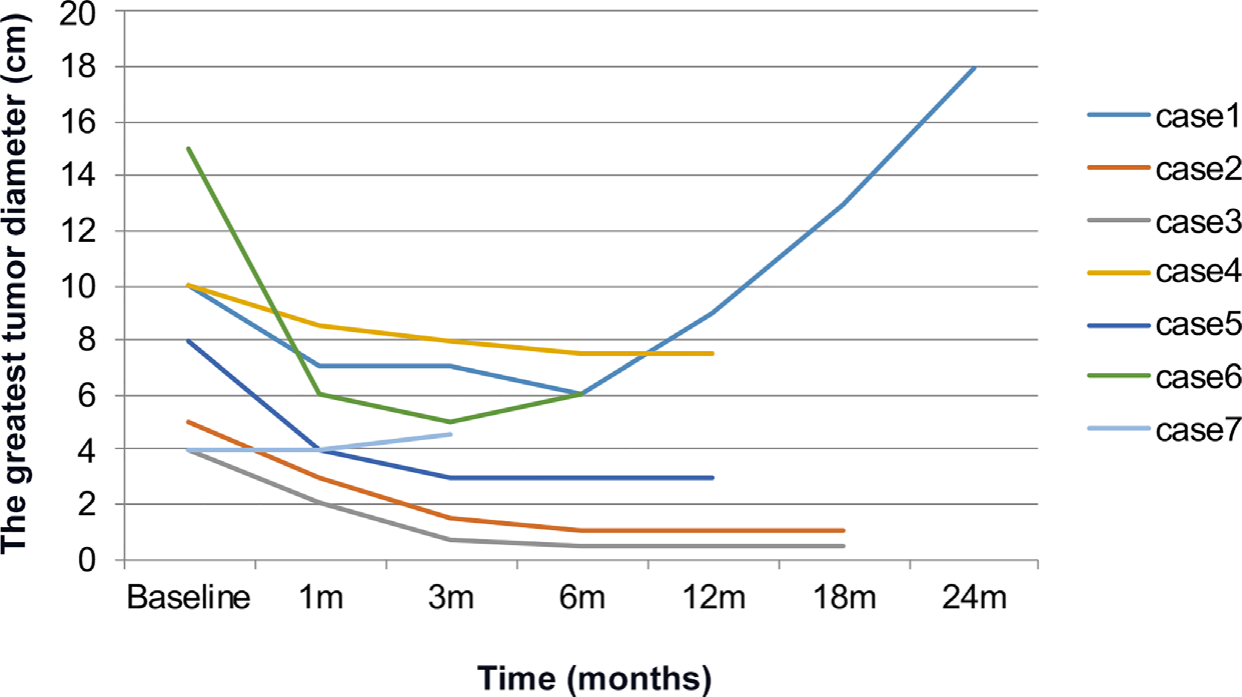
Changes of tumor diameter in patients treated with everolimus.

Seven patients were treated with everolimus 10 mg once daily for 3 to 28 months. The clinical response was evaluated after 1-month treatment: five PR, 2 SD, the mean longest tumor diameter of all patients decreased from 9.6 to 6.4 cm. After 3-month treatment: five PR, 2 SD, the mean greatest tumor diameter was 5.2 cm (**Figure 2**), and there was no significant change of tumor size at 6 months (**Figure 3**). After 3–28 months’ follow-up, six patients were SD; one patient suffered progression after 16 months and died after 26 months. The efficacy evaluation showed five patients with tuberous sclerosis were PR, in contrast, patients without tuberous sclerosis were SD.

The main adverse effects (AEs) including oral mucositis in five patients, rash in two patients, anemia in two patients, menstrual disorder in two patients and hepatic dysfunction in one patient. Most of the AEs were grade 1 or 2, no need to dosage reduction or discontinuation, and can be alleviated after symptomatic treatment. One patient had grade 3 liver dysfunction after 9 months’ treatment. The minimum blood concentration of rapamycin was 15.18 ng/ml, which converted to the blood concentration of everolimus was 19.7 ng/ml; it was relieved after everolimus suspension and symptomatic treatment. And then, this patient restarted everolimus treatment with an adjusted dose of 5 mg once daily. The blood concentration of everolimus was 7.4 ng/ml in re-examination.

## Discussion

Renal EAML is a rare renal neoplasm, represents less than 1% of all renal tumors, 4.6% in all kinds of renal angiomyolipoma (3). AMLs are benign and lack of specific clinical manifestations, when the lesions are small, most cases are asymptomatic and incidentally diagnosed. Renal EAMLs are always diagnosed with imaging examination, however, the imaging characteristics of renal EAML are diverse. Some tend to be more aggressive in appearance with invasion of adjacent organs or distant metastases, which lead to misdiagnosed as other types of renal tumors. In our study, the clinical presentation including pain in five patients and hematuria in one patient, MRI showed fat-deficient lesion. Two patients were diagnosed as tuberous sclerosis by clinical presentation.

Renal EAML has unique biological behaviors, predictors for malignant diagnosis and patient’s prognosis had been explored by other investigators. In 2015, Lei et al. have proposed predictive model based on tumor specimens and pathological features: 1) tumor size >9 cm, 2) tumor thrombus formation in the vein, 3) epithelioid cells >70% or atypia cells >60%, and 4) necrosis. A lesion that met three or more of the above features was predicted to have an increased risk of malignant behavior (12). Varma et al. have reported that P53-positive in renal EAML tumors indicating a tendency of malignant transformation, Ki67 and P53 staining were helpful in the distinguish of benign or malignant tumors (13).

In our study, all patients were HMB45 staining positive. Five of them had multiple metastases, their common characteristics included that the positive rate of Ki-67 was more than 10%, and the mitotic figure was more than 2/10 HPF. For the other two patients, the positive rate of Ki-67 was 3%, and the mitotic figure was 1–2/10HPF. A young female patient in our study developed tumor progression rapidly within a short period which inducing abortion. It is presumed that this phenomenon may be associated with high expression of estrogen and progesterone receptors in this disease (14). Tsai et al. have showed family tuberous sclerosis or tumors in other sites can be detected in more than half of renal EAML patients (15). The proportion of tuberous sclerosis in renal EMAL was significantly higher than AML. About 20–30% of young renal EAML patients were accompanied with tuberous sclerosis (16). All patients in our study underwent TSC1/2 gene test, four patients had mutations in pathogenic TSC1 or TSC2 genes, five patients met the clinical or genetic diagnostic criteria for tuberous sclerosis, accounting for 35.7% (5/14) of EAML patients diagnosed in our hospital during the same period.

There is no standard treatment for renal EAML, surgical resection is effective and the complete tumor resection can improve the outcome (17). Referring to selecting surgical methods, we should consider the size, site, invasion of surrounding tissues, and distant metastasis of the tumors comprehensively. According to our results, five patients experienced local recurrences which reflected the importance of complete resection. Some investigators have investigated the benefit of treatment with rapamycin, cyclophosphamide, and cisplatin for renal EAML; while some cases report showed that renal EAML was not sensitive to radiotherapy, chemotherapy, or molecular targeted therapy (18–20). Kenerson et al. (7) reported abnormal activation of mTOR pathway may contribute to renal EAML growth and progression. In some patients, mTOR inhibitors such as rapamycin and everolimus reduced the size of the tumors, suggested that mTOR inhibitors may be therapeutic option for EAML. In a prospective, randomized, multicenter clinical study (EXIST-2), 79 AML patients diagnosed TSC received 6-month treatment of everolimus, the proportion of renal AML patients achieving ≥50% reductions in everolimus cohort was 42%, and 0 in control cohort (21). However, the use of everolimus in invasive EAML has been reported only in some cases (22–24).

The tumor size of five patients in our trial, diagnosed as EAML with tuberous sclerosis, significantly decreased after everolimus treatment; only one suffered progression and died. Two without tuberous sclerosis did not have a significant change in tumor size. We chose 10mg/day as an initial dose of everolimus based on previous treatment for renal angiomyolipoma with tuberous sclerosis. Most of the AEs were of grade 1–2, the incidence of grade 3–4 AEs was quite low. Regular follow up for safety and plasma concentration can minimize the risk of serious AEs. Dosage adjustment can be considered based on the plasma concentration, which will be helpful for patients’ safety and compliance to treatment.

In conclusion, invasive renal EAML is a type of tumor with malignant potential, and the complete tumor resection is a key factor for cure. Gene mutation analysis of TSC1 and TSC2 should be performed in the patient whose lesion can’t be resected completely in preoperative assessment, or progressed after surgery. The mTOR inhibitor can be an effective treatment for patients with invasive malignant renal EAML. Patients with TSC may benefit more from the therapy. Our finding still needs to be confirmed in a larger, prospective, randomized trial.

## Data Availability Statement

The original contributions presented in the study are included in the article/**Supplementary Material**, further inquiries can be directed to the corresponding author.

## Ethics Statement

The studies involving human participants were reviewed and approved by Medical Ethics Committee of Chinese PLA General Hospital. Written informed consent to participate in this study was provided by the participants’ legal guardian/next of kin.

## Author Contributions

Conception and design: GG, LG, XZ. Data collection or management: GG, LG. data analysis: GG, LG. manuscript writing/editing: GG, LG, XZ. All authors contributed to the article and approved the submitted version.

## Conflict of Interest

The authors declare that the research was conducted in the absence of any commercial or financial relationships that could be construed as a potential conflict of interest.
