# Supplementary material for: Everolimus in Invasive Malignant Renal Epithelioid Angiomyolipoma
Source: Front Oncol. 2021 Jan 26;10:610858. doi: 10.3389/fonc.2020.610858 (PMC7870865; doi:10.3389/fonc.2020.610858)
Supplement: Supplementary file 1 [file Image_1.pdf]

Gene sequencing reports for all patients.

Case 1

华大医学 BGI·Dx　　**深圳华大临床检验中心**　　DX-SDP-B09 V1.2

遗传性肿瘤基因检测报告

样本信息							
到样日期	样本编号	样本类型	姓名	性别	年龄	送检医院	送检医生
2016-11-14	16B3108373	全血	衣东辉	男	34		
临床表现或家族史		确认患者；血管平滑肌脂肪瘤；无家族遗传病史。					

检测信息	
检测疾病编号	DX1415
疾病名称	遗传性肿瘤-血管平滑肌脂肪瘤
检测基因	*TSC1*，*TSC2*
检测方法	芯片捕获高通量测序

检测结果								
基因	参考序列	核苷酸变化/突变名称	氨基酸变化	基因亚区	杂合性	染色体位置	参考文献	变异类型
TSC1	NM_000368	c.1700C>T	p.Ala567Val	EX15/CDS13	Het	chr9:135781265	[1-2]	VUS

备注：**杂合性：Het表示杂合突变，Hom表示纯合突变，Hemi表示半合子突变。**变异类型：Pathogenic表示已知致病突变，Likely pathogen表示疑似致病突变，VUS表示临床意义未明突变，Likely benign表示疑似良性突变，Benign表示良性突变。

结果说明

本次检测，未发现受检者在检测范围内存在已知致病性突变。结合疾病的发病率、位点在各数据库中的频率及受检者临床主述，检出1个临床意义未明位点。

位点详情：*TSC1*; NM_000368; c.1700 C>T; p.Ala567Val; CDS13; Het：错义突变，已有该变异的相关文献报道，但致病性未确定，临床意义未明。其在正常人群中发生的概率较低。经Polyphen2和Mutation taster对其进行蛋白质功能预测，结果均为无害。

备注：以上解读基于目前对检测疾病致病基因的研究。检测疾病基因、检测方法及局限性、目标区域高通量测序参数、检出变异点见附录。

建议

建议医生结合临床进一步分析临床意义未明位点。

参考文献

[1] Hoogeveen-Westerveld M, Ekong R, Povey S, et al. Functional assessment of *TSC1* missense variants identified in individuals with tuberous sclerosis complex[J]. Human mutation, 2012, 33(3): 476-479.

[2] Zhang J, Walsh M F, Wu G, et al. Germline mutations in predisposition genes in pediatric cancer[J]. New England Journal of Medicine, 2015, 373(24): 2336-2346.

**本报告结果只对送检样品负责。本中心对以上检测结果保留最终解释权，如有疑义，请在收到结果后的7个工作日内与我们联系。
**以上结论均为实验室检测数据，仅用于突变检测之目的，不代表最终诊断结果，仅供临床参考。
**数据解读规则参考美国医学遗传学和基因组学学院（American College of Medical Genetics and Genomics，ACMG）相关指南。
**变异命名参照 HGVS建议的规则给出（http://www.hgvs.org/mutnomen/）。

实验操作人：徐秋徽　　报告撰写人：熊云　　审核人：张晓平　　报告日期：2016-12-02

深圳华大临床检验中心 报告专用章

地址：广东省深圳市盐田区北山工业区 11栋（邮编：518083）客服电话：400-605-6655网址：www.bgidx.cn

附录

1. 疾病检测基因

英文名称	中文名称	检测基因
Angiomyolipoma	血管平滑肌脂肪瘤	*TSC1,TSC2*

2. 检测方法与局限性

本方法以受检者血液、唾液或其他组织来源的基因组 DNA为检测材料，首先进行将 DNA打断并制备文库，然后通过芯片对目标基因编码区及临近剪切区的 DNA进行捕获和富集，最后使用高通量测序平台进行突变检测。本方法适用于点突变及 20bp以内的缺失插入突变（微小突变）以及外显子水平的纯合型缺失检测，不适用于杂合性基因大片段拷贝数变异、动态突变及复杂重组等特殊类型突变的检测，也不适用于检测基因组结构变异（例如大片段缺失、复制与倒位重排）、大片段杂合插入突变（如 *Alu*介导的插入）及位于基因调节区及深度内含子区的突变。另外，由于部分基因存在高重复低复杂度区域或假基因，以致检测不能完全覆盖其所有外显子区，但总体覆盖度可达 95%以上。

3. 目标区捕获高通量测序参数

样本编号	16B3108373
目标基因数	2
目标区长度（bp）	26579
目标区覆盖度	100.00%
目标区平均深度（X）	825.88
目标区平均深度>30X位点所占比例	100.00%

4. 检测结果相关附图

无

地址：广东省深圳市盐田区北山工业区 11栋（邮编：518083）客服电话：400-605-6655网址：www.bgidx.cn

5. 相关基因编码区及其邻近**±10bp**内含子区的变异位点

序号	基因	转录本	核苷酸改变	氨基酸改变	基因亚区	杂合性	染色体位置	**RS-**号	千人频率	本地频率	变异类型
1	*TSC1*	NM_000368	c.1726T>C	p.Leu576Leu	EX15/CDS13	Het	chr9:135781239	rs118203567	0.0192	C	-
2	*TSC1*	NM_000368	c.1700C>T	p.Ala567Val	EX15/CDS13	Het	chr9:135781265	rs397514880	0	A	VUS

注：杂合性：Hom表示纯合突变，Het杂合突变，Hemi表示半合子突变。
千人频率：千人计划中全部测序样本中关于此 SNP的频率信息。
本地频率：本地收集的大于 200正常人测序样本中关于此 SNP的频率信息，（本地频率：0-0.01为 A；0.01-0.05为 B（包含 0.01和 0.05）；0.05-1为 C）。
变异类型：Pathogenic表示已知致病突变，Likely pathogenic表示疑似致病突变，VUS表示临床意义未明突变，Likely benign表示疑似良性突变，Benign表示良性突变。
染色体位置：点突变或缺失插入突变所在染色体位置（外显子缺失重复类型的突变，该数据项为空）。

Case 2

（2）与临床用药可能相关的体细胞变异及关键胚系变异

基因变异及丰度[1]	临床意义	靶向药物（敏感性
TSC2 NM_000548.4<br>27 号外显子 p.G1001E 错义突变<br>c.3002G>A p.Gly1001Glu<br>丰度：87.0%	携带 *TSC2* 功能缺失性突变或拷贝数缺失的肿瘤可能对 MTOR 抑制剂敏感。	依维莫司（尚不明确<br>坦罗莫司（尚不明<br>西罗莫司（尚不明
TSC2 NM_000548.4<br>41 号外显子非移码缺失性突变<br>c.5238_5255del<br>p.His1746_Arg1751del<br>丰度：47.3%	携带 *TSC2* 功能缺失性突变或拷贝数缺失的肿瘤可能对 MTOR 抑制剂敏感。	依维莫司（尚不<br>坦罗莫司（尚不<br>西罗莫司（尚不
[illegible]		

Case 3

华大医学 BGI·Dx 深圳华大临床检验中心 DX-SDP-B09 V1.2

遗传性肿瘤基因检测报告

样本信息							
到样日期	样本编号	样本类型	姓名	性别	年龄	送检医院	送检医生
2016-11-14	16B3108377	全血	侯红伟	男	50		
临床表现或家族史	确认患者；血管平滑肌脂肪瘤。无家族遗传病史。						

检测信息	
检测疾病编号	DX1415
疾病名称	遗传性肿瘤-血管平滑肌脂肪瘤
检测基因	*TSC1*，*TSC2*
检测方法	芯片捕获高通量测序

检测结果

基因	参考序列	核苷酸变化/突变名称	氨基酸变化	基因亚区	杂合性	染色体位置	参考文献	变异类型
TSC2	NM_000548	c.1832G>A	p.Arg611Gln	EX17/CDS16	Het	chr16:2120572	[1-3]	Pathogenic

备注：**杂合性：Het表示杂合突变，Hom表示纯合突变，Hemi表示半合子突变。**变异类型：Pathogenic表示已知致病突变，Likely pathogen表示疑似致病突变，VUS表示临床意义未明突变，Likely benign表示疑似良性突变，Benign表示良性突变。

结果说明

本次检测，在受检者中检出*TSC2*基因的已知致病突变 c.1832G>A (p.Arg611Gln; Het)。*TSC2*基因相关的结节性硬化病为常染色体显性遗传，因此推测该突变可能导致疾病发生。

位点详情：*TSC2*; NM_000548; c.1832 G>A; p.Arg611Gln; CDS16; Het：错义突变，已有文献报道该位点的致病性。该位点在人群中发生频率极低。经 SIFT和 Mutation Taster对其进行蛋白功能预测，结果均为有害。

备注：以上解读基于目前对检测疾病致病基因的研究。检测疾病基因、检测方法及局限性、目标区域高通量测序参数、检出变异点见附录。

建议

建议受检者其他亲属进行家系验证并接受遗传咨询。

参考文献

[1] Au K S, Williams A T, Roach E S, et al. Genotype/phenotype correlation in 325 individuals referred for a diagnosis of tuberous sclerosis complex in the United States[J]. Genetics in Medicine, 2007, 9(2): 88-100.
[2] Ali M, Girimaji S C, Markandaya M, et al. Mutation and polymorphism analysis of *TSC1* and *TSC2* genes in Indian patients with tuberous sclerosis complex[J]. Acta Neurologica Scandinavica, 2005, 111(1): 54-63.
[3] Hung C C, Su Y N, Chien S C, et al. Molecular and clinical analyses of 84 patients with tuberous sclerosis complex[J]. BMC medical genetics, 2006, 7(1): 1.

**本报告结果只对送检样品负责。本中心对以上检测结果保留最终解释权，如有疑义，请在收到结果后的7个工作日内与我们联系。
**以上结论均为实验室检测数据，仅用于突变检测之目的，不代表最终诊断结果，仅供临床参考。
**数据解读规则参考美国医学遗传学和基因组学学院（American College of Medical Genetics and Genomics，ACMG）相关指南。
**变异命名参照 HGVS建议的规则给出（http://www.hgvs.org/mutnomen/）。

实验操作人：徐秋徽　　报告撰写人：熊云　　审核人：张晓平　　报告日期：2017-01-03

深圳华大临床检验中心 报告专用章

附录

1. 疾病检测基因

英文名称	中文名称	检测基因
Angiomyolipoma	血管平滑肌脂肪瘤	*TSC1,TSC2*

2. 检测方法与局限性

本方法以受检者血液、唾液或其他组织来源的基因组 DNA为检测材料，首先进行将 DNA打断并制备文库，然后通过芯片对目标基因编码区及临近剪切区的 DNA进行捕获和富集，最后使用高通量测序平台进行突变检测。本方法适用于点突变及 20bp以内的缺失插入突变（微小突变）以及外显子水平的纯合型缺失检测，不适用于杂合性基因大片段拷贝数变异、动态突变及复杂重组等特殊类型突变的检测，也不适用于检测基因组结构变异（例如大片段缺失、复制与倒位重排）、大片段杂合插入突变（如 *Alu*介导的插入）及位于基因调节区及深度内含子区的突变。另外，由于部分基因存在高重复低复杂度区域或假基因，以致检测不能完全覆盖其所有外显子区，但总体覆盖度可达 95%以上。

3. 目标区捕获高通量测序参数

样本编号	16B3108377
目标基因数	2
目标区长度（bp）	26579
目标区覆盖度	100.00%
目标区平均深度（X）	563.27
目标区平均深度>30X位点所占比例	99.45%

4. 检测结果相关附图

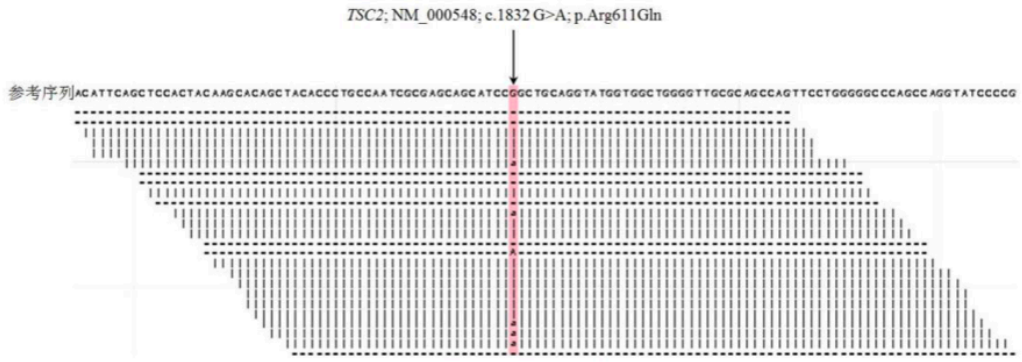

注：上图显示为正链reads结果

地址：广东省深圳市盐田区北山工业区 11栋（邮编：518083）客服电话：400-605-6655网址：www.bgidx.cn

5. 相关基因编码区及其邻近**±10bp**内含子区的变异位点

序号	基因	转录本	核苷酸改变	氨基酸改变	基因亚区	杂合性	染色体位置	**RS-**号	千人频率	本地频率	变异类型
1	*TSC2*	NM_000548	c.3475C>T	p.Arg1159Trp	EX30/CDS29	Het	chr16:2130243	rs45517295	0.0018	B	-
2	*TSC1*	NM_000368	c.1335A>G	p.Glu445Glu	EX14/CDS12	Het	chr9:135782221	rs7862221	0.2381	C	-
3	*TSC1*	NM_000368	c.965T>C	p.Met322Thr	EX10/CDS8	Het	chr9:135786904	rs1073123	0.2335	C	-
4	*TSC2*	NM_000548	c.1832G>A	p.Arg611Gln	EX17/CDS16	Het	chr16:2120572	rs28934872	0	A	Pathogenic

注：杂合性：Hom表示纯合突变，Het杂合突变，Hemi表示半合子突变。

千人频率：千人计划中全部测序样本中关于此 SNP的频率信息。

本地频率：本地收集的大于 200正常人测序样本中关于此 SNP的频率信息，（本地频率：0-0.01为 A；0.01-0.05为 B（包含 0.01和 0.05）；0.05-1为 C）。

变异类型：Pathogenic表示已知致病突变，Likely pathogenic表示疑似致病突变，VUS表示临床意义未明突变，Likely benign表示疑似良性突变，Benign表示良性突变。

染色体位置：点突变或缺失插入突变所在染色体位置（外显子缺失重复类型的突变，该数据项为空）。

地址：广东省深圳市盐田区北山工业区 11栋（邮编：518083）客服电话：400-605-6655网址：www.bgidx.cn

Case 4

漫瑞康诺基因检测中心

遗传肿瘤基因检测报告

样本信息						
送检日期	样本编号	姓名	年龄	性别	样本类型	样本体积
2017-10-17	M3-615	马秀芝	60	女	外周血	10ml
临床表现或有无家族史		左侧肾脏切除，临床表现良好				

检测信息	
检测疾病编号（报告编号）	LH-2003083
确证疾病名称	双侧肾错构瘤
检测基因名称	*TSC1*、*TSC2*
检测方法	特异性探针杂交捕获结合二代高通量测序

检测结果

基因	染色体位置及条带信息	突变区域	突变类型	参考序列	突变位点	氨基酸突变	纯合或杂合	参考文献	突变对疾病影响程度
TSC1	Chr9:135767943	UTR3	点突变	NM_000368.4	c.*3679G>A	NA	杂合	/	良性
TSC1	Ch9:135770115	UTR3	点突变	NM_000368.4	c.*1507G>A	NA	杂合	/	良性
TSC1	Chr9:135770134	UTR3	点突变	NM_000368.4	c.*1488C>T	NA	杂合	[1]	良性
TSC1	Ch:9:135770300	UTR3	点突变	NM_000368.4	c.*1322C>T	NA	杂合	/	良性
TSC1	Ch:9:135770347	UTR3	点突变	NM_000368.4	c.*1275T>G	NA	杂合	/	良性
TSC1	Ch:9:135771333	UTR3	点突变	NM_000368.4	c.*289delT	NA	杂合	/	良性
TSC1	Ch:9:135773000	Intronic	点突变	NM_000368.4	c.2626-4delTTTTTT	NA	杂合	/	良性

结果说明

本次检测在受检者样本中共检出了 7 个位点的变异，均发生在 TSC1 基因的 UTR3 区域，属于非结构变异。

TSC1 基因编码错构瘤蛋白，其具体功能仍不明确，能与 TSC2 基因编码的 tuberin 蛋白互相作用形成复合体，通过调节 Mtor 信号通路调节细胞生长。因此 TSC 基因变异可能扰乱细胞正常活动，诱发结节性硬化症，在不同的组织或器官促使肿瘤细胞的生长。结节性硬化症属于常染色体显性遗传病，这些位点对结节性硬化症的影响在 ClinVar（https://www.ncbinlm.nih.gov/clinvar/variation/65157/）数据库均有报道，但临床重要性并未完全确认。

综合建议

建议受检者结合本报告内容与主治医师进行沟通咨询！

参考文献

[1]Schaefer GB, Mendelsohn NJ; Professional Practice and Guidelines Committee.Clinical genetics evaluation in identifying the etiology of autism spectrum disorders: 2013 guideline revisions. Genet Med 2013 May;15(5):399-407. Epub 2013 Mar 21 doi: 10.1038/gim.2013.32. (PMID: 23519317)

漫瑞康诺基因检测中心

本报告只对送检样本负责，本报告及所使用的试剂及检测方法仅为科学研究目的，不能作为临床诊断或治疗的依据。其解释权归北京漫瑞诺康生物技术有限公司所有。

实验操作人：程薇 郝晓娟	报告撰写人：张腾龙	报告审核人：车建为

Case 5

华大医学 BGI·Dx　　深圳华大临床检验中心　　DX-SDP-B09 V1.2

遗传性肿瘤基因检测报告

样本信息							
到样日期	样本编号	样本类型	姓名	性别	年龄	送检医院	送检医生
2016-11-14	16B3108383	全血	管娜娜	女	25		
临床表现或家族史	确认患者；血管平滑肌脂肪瘤。无家族遗传病史。						

检测信息	
检测疾病编号	DX1415
疾病名称	遗传性肿瘤-血管平滑肌脂肪瘤
检测基因	*TSC1*，*TSC2*
检测方法	芯片捕获高通量测序

检测结果

基因	参考序列	核苷酸变化/突变名称	氨基酸变化	基因亚区	杂合性	染色体位置	参考文献	变异类型
-	-	-	-	-	-	-	-	-

备注：**杂合性：Het表示杂合突变，Hom表示纯合突变，Hemi表示半合子突变。**变异类型：Pathogenic表示已知致病突变，Likely pathogen表示疑似致病突变，VUS表示临床意义未明突变，Likely benign表示疑似良性突变，Benign表示良性突变。

结果说明

本次检测，未发现受检者在检测范围内存在已知致病性突变。所有检出位点信息见附录。

备注：以上解读基于目前对检测疾病致病基因的研究。检测疾病基因、检测方法及局限性、目标区域高通量测序参数、检出变异点见附录。

建议

检测结果为阴性并不能排除受检者患病可能，建议进行全基因组测序分析，或考虑其他具有相似临床表型的疾病。

参考文献

无

**本报告结果只对送检样品负责。本中心对以上检测结果保留最终解释权，如有疑义，请在收到结果后的7个工作日内与我们联系。
**以上结论均为实验室检测数据，仅用于突变检测之目的，不代表最终诊断结果，仅供临床参考。
**数据解读规则参考美国医学遗传学和基因组学学院（American College of Medical Genetics and Genomics，ACMG）相关指南。
**变异命名参照HGVS建议的规则给出（http://www.hgvs.org/mutnomen/）。

实验操作人：徐秋徽　　报告撰写人：熊云　　审核人：张晓平　　报告日期：2016-12-01

深圳华大临床检验中心 报告专用章

地址：广东省深圳市盐田区北山工业区11栋（邮编：518083）客服电话：400-605-6655网址：www.bgidx.cn

附录

1. 疾病检测基因

英文名称	中文名称	检测基因
Angiomyolipoma	血管平滑肌脂肪瘤	*TSC1,TSC2*

2. 检测方法与局限性

本方法以受检者血液、唾液或其他组织来源的基因组 DNA为检测材料，首先进行将 DNA打断并制备文库，然后通过芯片对目标基因编码区及临近剪切区的 DNA进行捕获和富集，最后使用高通量测序平台进行突变检测。本方法适用于点突变及 20bp以内的缺失插入突变（微小突变）以及外显子水平的纯合型缺失检测，不适用于杂合性基因大片段拷贝数变异、动态突变及复杂重组等特殊类型突变的检测，也不适用于检测基因组结构变异（例如大片段缺失、复制与倒位重排）、大片段杂合插入突变（如 *Alu*介导的插入）及位于基因调节区及深度内含子区的突变。另外，由于部分基因存在高重复低复杂度区域或假基因，以致检测不能完全覆盖其所有外显子区，但总体覆盖度可达 95%以上。

3. 目标区捕获高通量测序参数

样本编号	16B3108383
目标基因数	2
目标区长度（bp）	26579
目标区覆盖度	100.00%
目标区平均深度（X）	699.64
目标区平均深度>30X位点所占比例	100.00%

4. 检测结果相关附图

无

地址：广东省深圳市盐田区北山工业区 11栋（邮编：518083）客服电话：400-605-6655网址：www.bgidx.cn

5. 相关基因编码区及其邻近**±10bp**内含子区的变异位点

序号	基因	转录本	核苷酸改变	氨基酸改变	基因亚区	杂合性	染色体位置	**RS-**号	千人频率	本地频率	变异类型
-	-	-	-	-	-	-	-	-	-	-	-

注：杂合性：Hom表示纯合突变，Het杂合突变，Hemi表示半合子突变。
千人频率：千人计划中全部测序样本中关于此 SNP的频率信息。
本地频率：本地收集的大于 200正常人测序样本中关于此 SNP的频率信息，（本地频率：0-0.01为 A；0.01-0.05为 B（包含 0.01和 0.05）；0.05-1为 C）。
变异类型：Pathogenic表示已知致病突变，Likely pathogenic表示疑似致病突变，VUS表示临床意义未明突变，Likely benign表示疑似良性突变，Benign表示良性突变。
染色体位置：点突变或缺失插入突变所在染色体位置（外显子缺失重复类型的突变，该数据项为空）。

地址：广东省深圳市盐田区北山工业区 11栋（邮编：518083）客服电话：400-605-6655网址：www.bgidx.cn

Case 6

华大医学 BGI·Dx 深圳华大临床检验中心 DX-SDP-B09 V1.2

遗传性肿瘤基因检测报告

样本信息							
到样日期	样本编号	样本类型	姓名	性别	年龄	送检医院	送检医生
2016-12-16	16B0025303	全血	彭滔	-	-		
临床表现或家族史		确认患者；TSC，血管平滑肌脂肪瘤。无家族遗传病史。					

检测信息	
检测疾病编号	DX1415
疾病名称	遗传性肿瘤-血管平滑肌脂肪瘤
检测基因	*TSC1*，*TSC2*
检测方法	芯片捕获高通量测序

检测结果

基因	参考序列	核苷酸变化/突变名称	氨基酸变化	基因亚区	杂合性	染色体位置	参考文献	变异类型
TSC2	NM_000548	EX2_3DEL	-	EX2_3	Het	-	-	Likely pathogenic

备注：**杂合性：Het表示杂合突变，Hom表示纯合突变，Hemi表示半合子突变。**变异类型：Pathogenic表示已知致病突变，Likely pathogeni 表示疑似致病突变，VUS表示临床意义未明突变，Likely benign表示疑似良性突变，Benign表示良性突变。

结果说明

本次检测，在受检者中检出*TSC2*基因的杂合疑似致病突变EX2_EX3 DEL。*TSC2*基因相关的结节性硬化病为常染色体显性遗传，因此推测该突变可能导致疾病发生。

位点详情：*TSC2*; NM_000548; EX2_EX3 DEL; Het：大片段杂合缺失突变，暂未发现该片段的致病性的相关文献报道。

备注：以上解读基于目前对检测疾病致病基因的研究。检测疾病基因、检测方法及局限性、目标区域高通量测序参数、检出变异点见附录。

建议

建议受检者其他亲属进行家系验证并接受遗传咨询。

参考文献

无

**本报告结果只对送检样品负责。本中心对以上检测结果保留最终解释权，如有疑义，请在收到结果后的7个工作日内与我们联系。
**以上结论均为实验室检测数据，仅用于突变检测之目的，不代表最终诊断结果，仅供临床参考。
**数据解读规则参考美国医学遗传学和基因组学学院（American College of Medical Genetics and Genomics，ACMG）相关指南。
**变异命名参照 HGVS建议的规则给出（http://www.hgvs.org/mutnomen/）。

实验操作人：徐秋徽　　报告撰写人：熊云　　审核人：张晓平　　报告日期：2017-01-10

深圳华大临床检验中心 报告专用章

地址：广东省深圳市盐田区北山工业区 11栋（邮编：518083）客服电话：400-605-6655网址：www.bgidx.cn

附录

1. 疾病检测基因

英文名称	中文名称	检测基因
Angiomyolipoma	血管平滑肌脂肪瘤	*TSC1,TSC2*

2. 检测方法与局限性

本方法以受检者血液、唾液或其他组织来源的基因组 DNA为检测材料，首先进行将 DNA打断并制备文库，然后通过芯片对目标基因编码区及临近剪切区的 DNA进行捕获和富集，最后使用高通量测序平台进行突变检测。本方法适用于点突变及 20bp以内的缺失插入突变（微小突变）以及外显子水平的纯合型缺失检测，不适用于杂合性基因大片段拷贝数变异、动态突变及复杂重组等特殊类型突变的检测，也不适用于检测基因组结构变异（例如大片段缺失、复制与倒位重排）、大片段杂合插入突变（如 *Alu*介导的插入）及位于基因调节区及深度内含子区的突变。另外，由于部分基因存在高重复低复杂度区域或假基因，以致检测不能完全覆盖其所有外显子区，但总体覆盖度可达 95%以上。

3. 目标区捕获高通量测序参数

样本编号	16B0025303
目标基因数	2
目标区长度（bp)	26579
目标区覆盖度	100.00%
目标区平均深度（X)	446.58
目标区平均深度>30X位点所占比例	99.76%

4. 检测结果相关附图

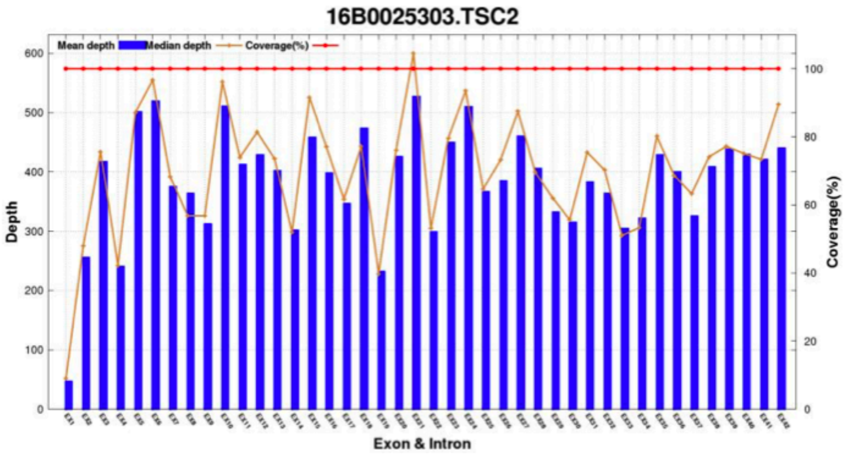

地址：广东省深圳市盐田区北山工业区 11栋（邮编：518083）客服电话：400-605-6655网址：www.bgidx.cn

5. 相关基因编码区及其邻近**±10bp**内含子区的变异位点

序号	基因	转录本	核苷酸改变	氨基酸改变	基因亚区	杂合性	染色体位置	**RS-**号	千人频率	本地频率	变异类型
1	*TSC2*	NM_000548	c.856A>G	p.Met286Val	EX10/CDS9	Het	chr16:2108755	rs1800748	0.0211	B	-
2	*TSC2*	NM_000548	c.3421G>A	p.Ala1141Thr	EX30/CDS29	Het	chr16:2130189	rs45505895	0	A	-
3	*TSC1*	NM_000368	c.1960C>G	p.Gln654Glu	EX15/CDS13	Het	chr9:135781005	rs75820036	0.0092	A	-
4	*TSC2*	NM_000548	EX2_3DEL	-	EX2_3	Het	-	-	-	-	Likely pathogenic

注：杂合性：Hom表示纯合突变，Het杂合突变，Hemi表示半合子突变。
千人频率：千人计划中全部测序样本中关于此 SNP的频率信息。
本地频率：本地收集的大于 200正常人测序样本中关于此 SNP的频率信息，（本地频率：0-0.01为 A；0.01-0.05为 B（包含 0.01和 0.05）；0.05-1为 C）。
变异类型：Pathogenic表示已知致病突变，Likely pathogenic表示疑似致病突变，VUS表示临床意义未明突变，Likely benign表示疑似良性突变，Benign表示良性突变。
染色体位置：点突变或缺失插入突变所在染色体位置（外显子缺失重复类型的突变，该数据项为空）。

地址：广东省深圳市盐田区北山工业区 11栋（邮编：518083）客服电话：400-605-6655网址：www.bgidx.cn

Case 7

华大医学 BGI·Dx　　**深圳华大临床检验中心**　　DX-SDP-B09 V1.2

遗传性肿瘤基因检测报告

样本信息							
到样日期	样本编号	样本类型	姓名	性别	年龄	送检医院	送检医生
2017-03-19	17B0015673	全血	张纯	-	-		-
临床表现或家族史	确认患者；血管平滑肌脂肪瘤；无家族史。						

检测信息	
检测疾病编号	DX1415
疾病名称	遗传性肿瘤-血管平滑肌脂肪瘤
检测基因	*TSC1*，*TSC2*
检测方法	芯片捕获高通量测序

检测结果

基因	参考序列	核苷酸变化/突变名称	氨基酸变化	基因亚区	杂合性	染色体位置	参考文献	变异类型
-	-	-	-	-	-	-	-	-

备注：**杂合性：Het表示杂合突变，Hom表示纯合突变，Hemi表示半合子突变。**变异类型：Pathogenic表示已知致病突变，Likely pathogen表示疑似致病突变，VUS表示临床意义未明突变，Likely benign表示疑似良性突变，Benign表示良性突变。

结果说明

本次检测，未发现受检者在检测范围内存在已知或疑似致病性突变。所有检出位点信息见附录。

备注：以上解读基于目前对检测疾病致病基因的研究。检测疾病基因、检测方法及局限性、目标区域高通量测序参数、检出变异点见附录。

建议

检测结果为阴性并不能排除受检者患病可能，建议进行全基因组测序分析，或考虑其他具有相似临床表型的疾病。

参考文献

无

**本报告结果只对送检样品负责。本中心对以上检测结果保留最终解释权，如有疑义，请在收到结果后的7个工作日内与我们联系。
**以上结论均为实验室检测数据，仅用于突变检测之目的，不代表最终诊断结果，仅供临床参考。
**数据解读规则参考美国医学遗传学和基因组学学院（American College of Medical Genetics and Genomics，ACMG）相关指南。
**变异命名参照 HGVS建议的规则给出（http://www.hgvs.org/mutnomen/）。

实验操作人：宋海峰　　报告撰写人：熊云　　审核人：张晓平　　报告日期：2017-04-06

深圳华大临床检验中心 报告专用章

地址：广东省深圳市盐田区北山工业区11栋（邮编：518083）客服电话：400-605-6655网址：www.bgidx.cn

附录

1. 疾病检测基因

英文名称	中文名称	检测基因
Angiomyolipoma	血管平滑肌脂肪瘤	*TSC1, TSC2*

2. 检测方法与局限性

本方法以受检者血液、唾液或其他组织来源的基因组 DNA为检测材料，首先进行将 DNA打断并制备文库，然后通过芯片对目标基因编码区及临近剪切区的 DNA进行捕获和富集，最后使用高通量测序平台进行突变检测。本方法适用于点突变及 20bp以内的缺失插入突变（微小突变）以及外显子水平的纯合型缺失检测，不适用于杂合性基因大片段拷贝数变异、动态突变及复杂重组等特殊类型突变的检测，也不适用于检测基因组结构变异（例如大片段缺失、复制与倒位重排）、大片段杂合插入突变（如 *Alu*介导的插入）及位于基因调节区及深度内含子区的突变。另外，由于部分基因存在高重复低复杂度区域或假基因，以致检测不能完全覆盖其所有外显子区，但总体覆盖度可达 95%以上。

3. 目标区捕获高通量测序参数

样本编号	17B0015673
目标基因数	2
目标区长度（bp）	26579
目标区覆盖度	100.00%
目标区平均深度（X）	448.9
目标区平均深度>30X位点所占比例	100.00%

4. 检测结果相关附图

无

地址：广东省深圳市盐田区北山工业区 11栋（邮编：518083）客服电话：400-605-6655网址：www.bgidx.cn

5. 相关基因编码区及其邻近**±10bp**内含子区的变异位点

序号	基因	转录本	核苷酸改变	氨基酸改变	基因亚区	杂合性	染色体位置	**RS-**号	千人频率	本地频率	变异类型
1	*TSC1*	NM_000368	c.1960C>G	p.Gln654Glu	EX15/CDS13	Het	chr9:135781005	rs75820036	0.0092	A	-

注：杂合性：Hom表示纯合突变，Het杂合突变，Hemi表示半合子突变。
千人频率：千人计划中全部测序样本中关于此 SNP的频率信息。
本地频率：本地收集的大于 200正常人测序样本中关于此 SNP的频率信息，（本地频率：0-0.01为 A；0.01-0.05为 B（包含 0.01和 0.05）；0.05-1为 C）。
变异类型：Pathogenic表示已知致病突变，Likely pathogenic表示疑似致病突变，VUS表示临床意义未明突变，Likely benign表示疑似良性突变，Benign表示良性突变。
染色体位置：点突变或缺失插入突变所在染色体位置（外显子缺失重复类型的突变，该数据项为空）。

地址：广东省深圳市盐田区北山工业区 11栋（邮编：518083）客服电话：400-605-6655网址：www.bgidx.cn
